# Multiple Model Evaluation of the Masseteric-to-Facial Nerve Transfer for Reanimation of the Paralyzed Face and Quick Prognostic Prediction

**DOI:** 10.3389/fsurg.2022.735231

**Published:** 2022-03-15

**Authors:** Tengfei Li, Yanhui Liu, Shuxin Zhang, Wanchun Yang, Mingrong Zuo, Xuesong Liu

**Affiliations:** Department of Neurosurgery, West China Hospital, Sichuan University, Chengdu, China

**Keywords:** facial paralysis, masseteric-to-facial nerve transfer, FNGS2.0, Sunnybrook FGS, FaCE Scale, facial measurement

## Abstract

Facial paralysis is negatively associated with functional, aesthetic, and psychosocial consequences. The masseteric-to-facial nerve transfer (MFNT) has many advantages in facial reanimation. The aim is to evaluate the effectiveness of our MFNT technique and define the potential factors predictive of outcome. The authors conducted a retrospective review of 20 consecutive patients who underwent MFNT using the temporofacial trunk of facial nerve. Videotapes and images were documented and evaluated according to Facial Nerve Grading Scale 2.0 (FNGS2.0) and Sunnybrook Facial Grading System (FGS). The quality-of-life was obtained using the Facial Clinimetric Evaluation (FaCE) Scale. Moreover, Facial Asymmetry Index (FAI), quantitative measurement of the width of palpebral fissure, deviation of the philtrum, and angles or excursions of the oral commissure were applied to explore the effect of the transfer metrically. Multivariable logistic regression models and Cox regression were prepared to predict the effect of MFNT by preoperative clinical features. The patients showed favorable outcomes graded by FNGS2.0, and experienced significantly improved scores in static and dynamic symmetry with slightly elevated scores in synkinesis evaluated by the Sunnybrook FGS. The score of FaCE Scale increased in all domains after reanimation. The quantitative indices indicated the symmetry restoration of the middle and lower face after MFNT. Regression analysis revealed that younger patients with severe facial paralysis are preferable to receive MFNT early for faster and better recovery, especially for traumatic causes. The findings demonstrate that MFNT is an effective technique for facial reanimation, and case screening based on clinical characteristics could be useful for surgical recommendation.

## Background

Facial expression is a complex neuromotor and psychosocial process linking physical expression with emotion, responsible for essential functions such as eyelid closure, oral competence, and phonation of labial sounds, and provides a social participant with important clues to one's meanings, intentions, and emotional status. Correspondingly, facial nerve palsy is negatively associated with functional, aesthetic, and psychosocial consequences (1–3). In addition to leading to physical detriments including facial asymmetry, corneal ulceration, oral incompetence such as difficulties in eating, drinking, and speaking, facial paralysis causes psychosocial stress, decreased self-esteem, anxiety, depression, and social isolation (2, 4–7).

Facial reanimation surgery aims to restore resting symmetry, along with oral competence, eye closure, voluntary facial movement, and effortless spontaneous expression in the absence of synkinesis, while causing minimal loss of function due to harvesting of donor nerves. The facial symmetry and movement can be restored with different methods such as direct neurotization, nerve grafting, free muscle transfer, or various combined surgery. Adjacent nerve transfers are effective to reanimate the paralyzed face in cases with intact distal facial nerve branches as well as viable mimetic muscles. This situation frequently arises after tumor resection or trauma involving the skull base. A series of donor nerves have been utilized for facial reanimation with varying success including the contralateral facial nerve with cross-facial grafts, hypoglossal nerve, spinal accessory nerve, and the great auricular nerve. The power of motor source was thought to be the predominant cause of the disability in facial recovery after reanimation. Previous studies revealed that both the hypoglossal nerve and the masseteric nerve of the trigeminus are more powerful motor sources than contralateral facial cross nerve (8, 9), as a result, symmetry improvement and adequate excursion of the oral commissure can be realized easily by single motor source. Although direct hypoglossal-to-facial neurotization was once the most widely used technique, donor nerve-associated sequelae often yield unsatisfactory outcomes, such as tongue atrophy, speech and swallowing difficulty, mass movement, and synkinesis (10).

Spira et al. first described the role of the masseteric nerve in facial reanimation in 1978 (11). Subsequently, many researchers demonstrated the advantages of employing the masseteric nerve (8, 12–19). As a result, the masseteric nerve has recently emerged as a popular option for reanimating the paralyzed face, because it provides a large axonal load resulting in powerful muscle contraction and preferable symmetry, and it has the advantage of favorable proximity, faster onset of functional recovery, and lower risk of synkinesis (8, 12, 14, 16, 20–22). Particularly, the masseteric-to-facial nerve transfer (MFNT) avoids morbidity from nerve graft harvesting (14, 18, 23). Although the MFNT is widely applied in facial reanimation, the systematic evaluation of the efficacy and applicability of MFNT is limited.

In the present study, we describe our experience in 20 cases with improved MFNT technique for facial reanimation. Objective and subjective outcomes were quantified to evaluate the efficacy of the procedure, as well as to define the potential factors predictive of outcome preoperatively.

## Methods

### Study Design and Participants Characteristics

This study was approved by the Institutional Review Board of West China Hospital of Sichuan University and all patients provided written informed consent for record and publication of their figures and videos for research purposes. Between June 2018 and August 2020, 22 consecutive patients with facial paralysis underwent facial reanimation with the MFNT at West China Hospital by the senior author (X.S., L.). Of these, two patients were excluded due to the lack of long enough follow-up. Videotapes and images were documented as participants attempted to perform a series of tasks including resting, eyebrow raising, eye closure, snarl, and wide smile. Two independent physical therapists not involved in the surgery evaluated and graded the facial paralysis according to the Facial Nerve Grading Scale 2.0 (FNGS2.0) and Sunnybrook Facial Grading System (FGS) (24, 25). The authors translated the Facial Clinimetric Evaluation (FaCE) Scale into Chinese and administered it in patients preoperatively and postoperatively, then the scores were tabulated for comparison (26). Moreover, Facial Asymmetry Index (FAI) and quantitative measurement of the width of palpebral fissure, deviation of the philtrum, and angles or three directional excursions of the oral commissure were applied to explore the change after nerve transfer metrically (22).

### Surgical Technique

**Figure 1:**
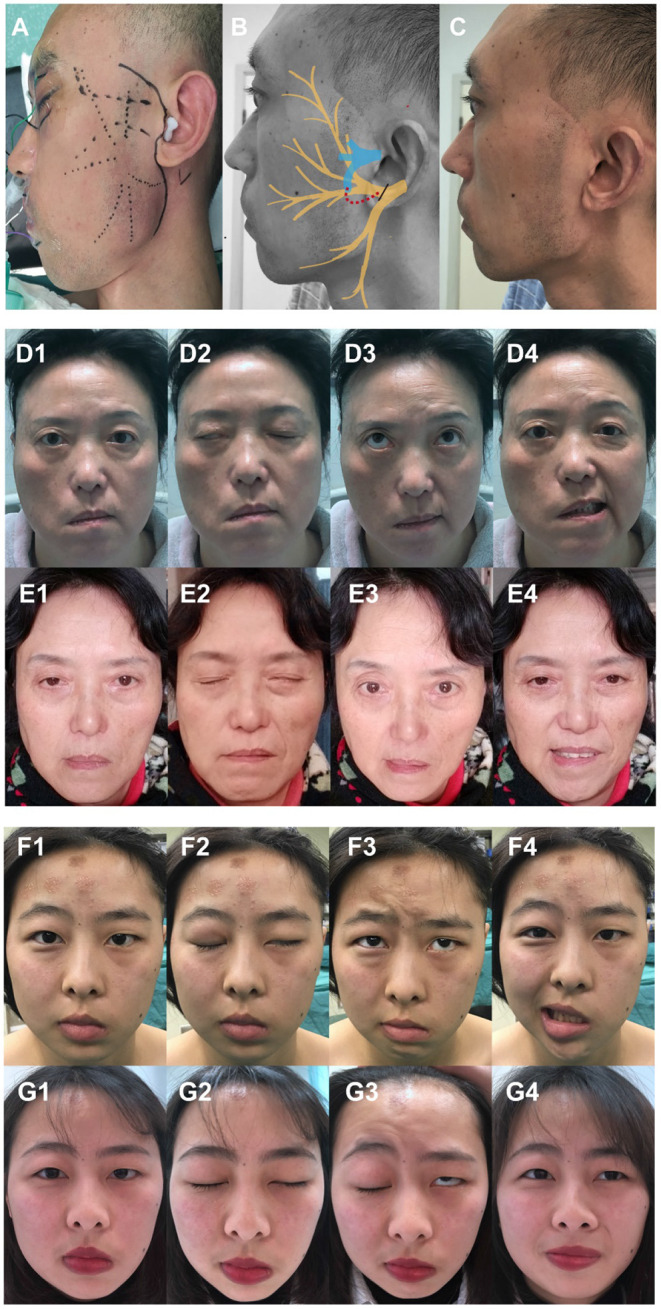
Surgical diagram illustrating the masseteric-to-facial nerve transfer (MFNT) using the temporofacial trunk of the facial nerve and representative pictures of two patients who underwent the MFNT at rest, eye close, frown, and smile. **(A)** Preauricular skin incision extending anterosuperiorly into the temporal region and inferiorly to 1 cm inferior to the angle of mandible. **(B)** Transfer of masseteric nerve to temporofacial trunk of facial nerve in an end-to-end manner. The main trunk of the facial nerve bifurcates into the temporofacial and cervicofacial trunks, and the former ramifies the frontal, zygomatic, and buccal branches consistently. The facial nerve is illustrated in yellow, the masseteric nerve in blue. Black short line indicates the severed facial nerve and dash line denotes direct anastomosis. **(C)** The incision healing after a well-hidden aesthetic suture. **(D,E)** Patient No.19, a 54-year-old female, suffered facial paralysis for 21 months due to the acoustic neurinoma and relative surgical complications. **(F,G)** Patient No.3, an 18-year-old female, suffered facial paralysis for 39 months because of the epidermoid cysts in the cerebellopontine angle and relative surgical complications.

The procedure was initiated by executing a preauricular skin incision, extending anterosuperiorly into the temporal region and inferiorly to 1 cm inferior to the mandibular angle (Figure 1A), and then elevating a flap to the anterior border of the parotid gland. A consistent starting point for dissection of the masseteric nerve was found 3 cm anterior to the tragus and 1 cm inferior to the zygomatic arch (27). The subzygomatic triangle is a constant anatomic landmark for rapid, reliable, and minimally invasive identification of the masseteric nerve (28). Blunt dissection and frequent probing with a nerve stimulator (Boston Medical Products, USA) were used during the deep muscular dissection until the motor branch of the trigeminal nerve was identified. The full length of the dominant descending branch of the masseteric nerve was divided carefully as far as possible to preserve the proximal branches and obtain adequate length of nerve; then, it was severed at the distal point. Anatomically, the main trunk of the facial nerve was traced into the parotid gland bifurcating into temporofacial and cervicofacial trunks, and the former ramifies the frontal, zygomatic, and buccal branches consistently. Dissection of the superior trunk was carried out proximally enough to allow subsequent tension-free neurorrhaphy to masseteric nerve. Finally, the masseteric nerve was coapted to the temporofacial trunk in an end-to-end manner using 8 10-0 nylon sutures in the nerve sheath (Figure 1B). Restoration of parotid fascia and meticulous hemostasis was mandatory and a well-hidden aesthetic suture was placed at the end of the surgery (Figure 1C).

### Rehabilitation Protocol

All patients were asked to maintain a soft diet for 4 weeks after surgery, then, biofeedback rehabilitation protocol was recommended consisting of smiling in front of a mirror. After 2–4 months of exercising, the patients were trained to smile without clenching teeth, and practice their smiles with family, friends, and eventually, with strangers. Figures 1D–G shows the representative pictures of two patients before and after operation.

### Multimodal Facial Nerve Evaluation

**Figure 2:**
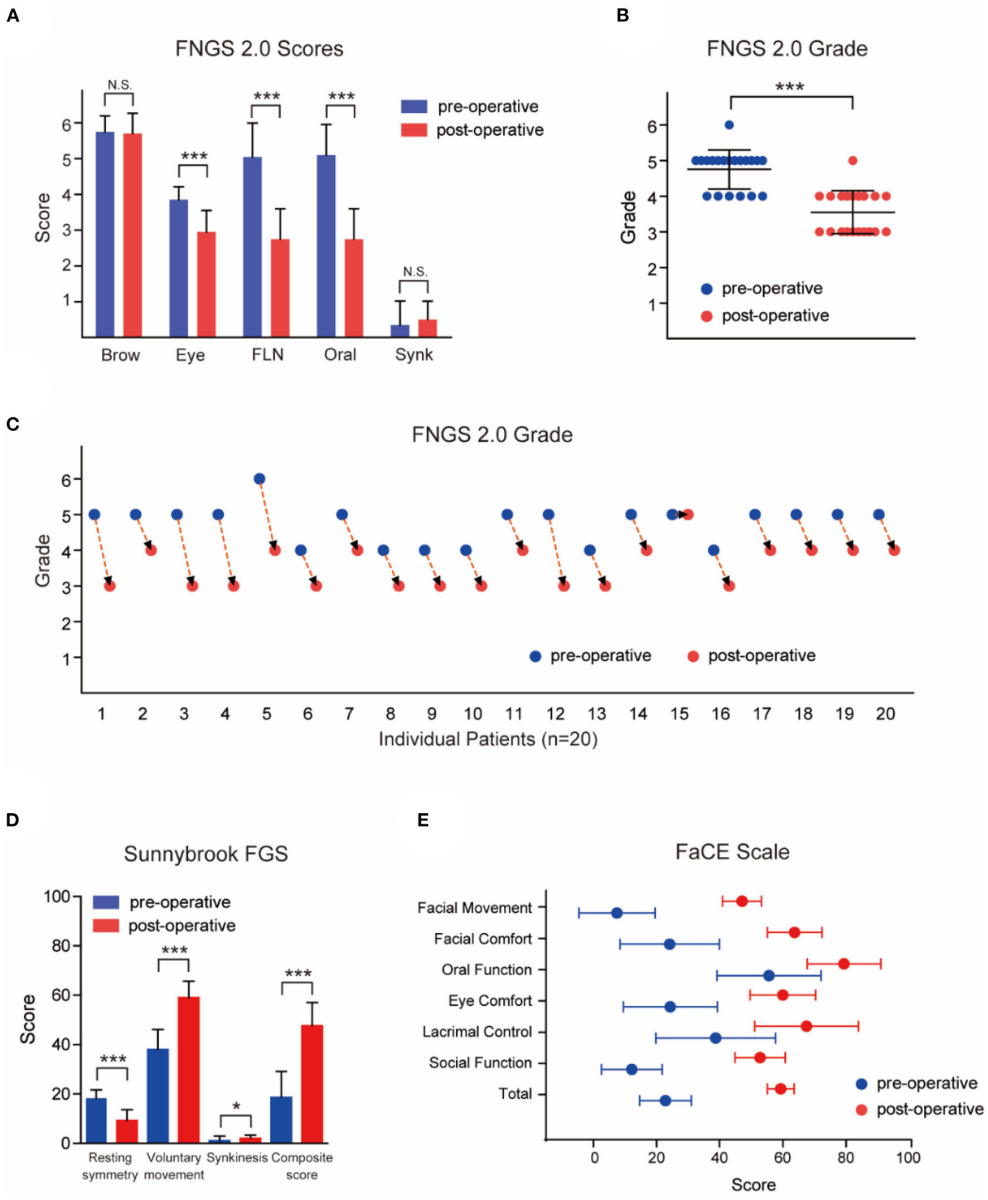
Outcomes of patients evaluated and graded according to Facial Nerve Grading Scale 2.0 (FNGS2.0), Sunnybrook Facial Grading System (FGS) and Facial Clinimetric Evaluation (FaCE) Scale. **(A)** The scores in eye close, nasolabial fold (FLN), and oral commissure subdomains decreased significantly after the transfer procedures (*P* < 0.001), while the synkinesis (Synk) score was not increased distinctly. **(B)** Patients achieved a preferable facial improvement after the reanimation (*P* < 0.001). **(C)** The grade deceased in 19 individual patients after surgery. **(D)** Patients experienced a significant improvement in resting symmetry domain and voluntary movement domain (both *P* < 0.001), but elevated score in synkinesis domain (*P* < 0.05). The median total composite score increased from 18 (12.5) to 49 (13.5) after the MFNT. **(E)** The median total score of the FaCE Scale improved significantly from 24.15 (14.58) to 59.15 (7.88) after reanimation with scores of all domains elevated. N.S. no statistical differences; *P < 0.05; ***P < 0.001.

Facial paralysis is a notoriously difficult problem to describe, rate, measure, and follow longitudinally. Although multiple scales have been developed and validated, unfortunately, there still exists no ideal method of rapid, effective, and consistent facial function scales (29, 30). The House-Brackmann scale, modified as FNGS2.0, and the Sunnybrook FGS are most widely used, but these assessments do not capture the patients' psychosocial well-being and perception of their faces. In the current practice, the emerging trend is to perform comprehensive assessments that include clinician-graded scales, patient-reported instruments, and quantitative measurements, or even layperson assessments of disfigurement. The results of three facial evaluation scales are shown in Table 1 and Figure 2.

**Table 1:** Scores of Facial Nerve Grading Scale 2.0 (FNGS2.0), Sunnybrook Facial Grading System (FGS) and Facial Clinimetric Evaluation (FaCE) scale for 20 patients before and after the masseteric-to-facial nerve transfer (MFNT).

**Scales**	**Median preoperative score (IQR)**	**Median postoperative score (IQR)**	***P* value**
**FNGS2.0**			
Brow (1–6)	6 (0.75)	6 (0.75)	0.317
Eye (1–6)	4 (0)	3 (0)	0.000
Nasolabial fold (1–6)	5 (1)	3 (1)	0.000
Oral commissure (1–6)	5 (1)	3 (1)	0.000
Synkinesis (0–3)	0 (0.75)	0.5 (1)	0.257
**Total movement**	20 (2.75)	14.5 (1.75)	0.000
**Final grade**	5 (1)	3.5 (1)	0.000
**Sunnybrook FGS**			
**Resting symmetry (0–1/2)**			
Eye (0–1)	1 (0)	1 (1)	0.014
Cheek-NLF (0–2)	2 (0)	1 (0)	0.000
Mouth (0–1)	1 (0)	0 (1)	0.001
Total resting symmetry score (0–20)	20 (3.75)	10 (8.75)	0.000
**Symmetry of voluntary movement (1–5)**			
Brow lift	1 (0)	1 (0)	1.000
Gentle eye closure	3 (0)	4 (1)	0.001
Open mouth smile	2 (1)	3 (0.75)	0.000
Snarl	2 (1)	3 (1)	0.000
Lip pucker	2 (1)	4 (1)	0.000
Total voluntary movement score (20–100)	40 (11)	60 (8)	0.000
**Synkinesis (0–3)**			
Brow lift	0 (0)	0 (0)	0.317
Gentle eye closure	0 (0)	0 (0)	0.317
Open mouth smile	0 (1)	1 (0)	0.007
Snarl	0 (1)	1 (0)	0.033
Lip pucker	0 (0)	0 (0.75)	0.564
Total synkinesis score (0–15)	0 (1.75)	2 (2)	0.024
**Total composite score**	18 (12.5)	49 (13.5)	0.000
**FaCE Scale**			
Facial Movement Score	0 (14.6)	50 (8.3)	0.000
Facial Comfort Score	25 (29.2)	66.7 (14.63)	0.000
Oral Function Score	62.5 (21.88)	81.25 (12.5)	0.000
Eye Comfort Score	25 (25)	62.5 (21.88)	0.000
Lacrimal Control Score	37.5 (25)	75 (25)	0.000
Social Function Score	12.5 (17.23)	50 (17.15)	0.000
**Total Score**	24.15 (14.58)	59.15 (7.88)	0.000

### Restoration of Facial Symmetry

**Figure 3:**
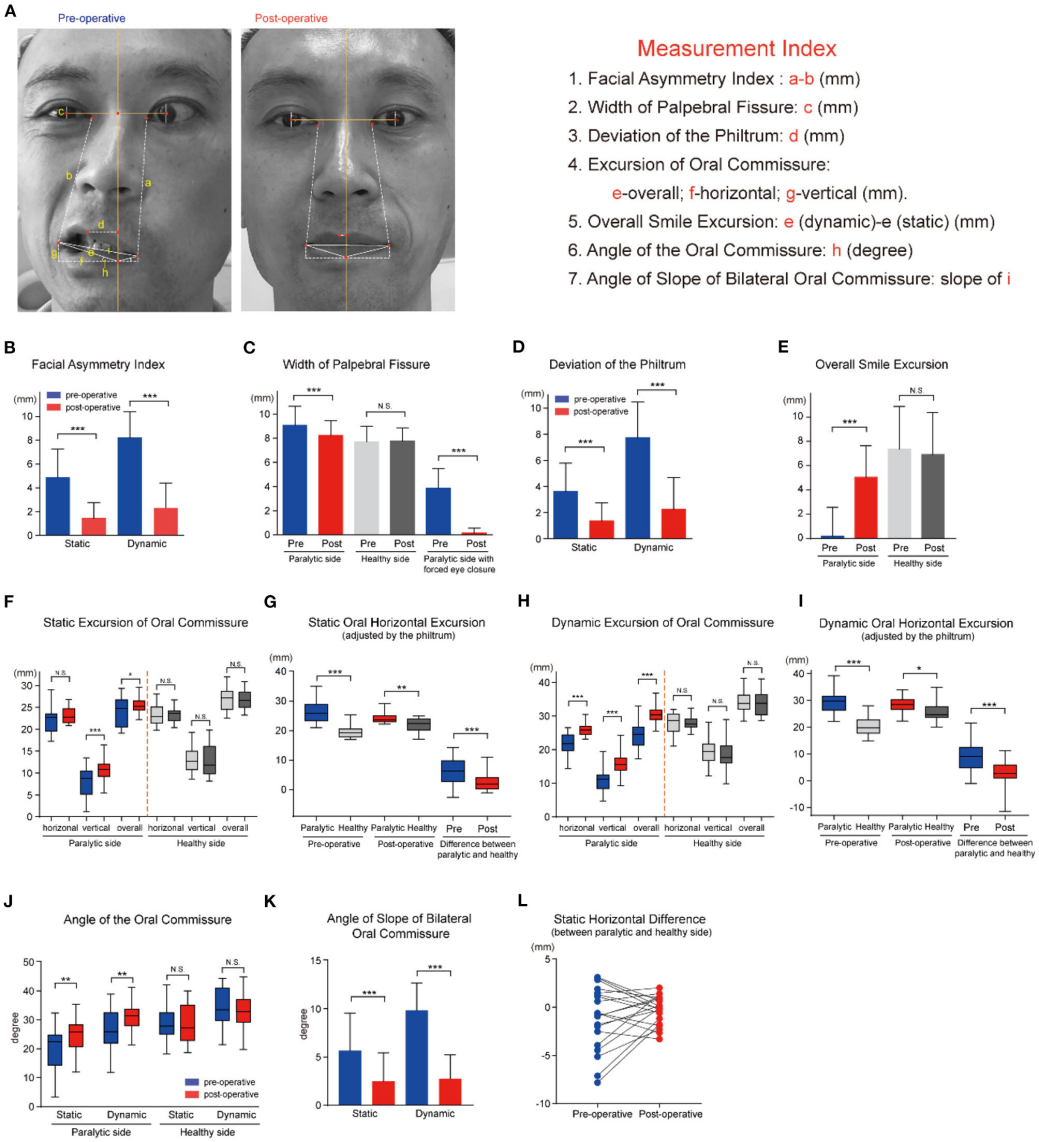
Multimodal facial measurement system to evaluate the effectiveness of the MFNT for facial reanimation. **(A)** Schemata of measurement indices of facial symmetry. **(B)** The Facial Asymmetry Index (FAI) was 4.87 mm at rest preoperatively, and significantly reduced to 1.46 mm postoperatively (*P* < 0.001). Furthermore, the FAI with smile decreased from 8.21 mm to 2.32 mm (*P* < 0.001). **(C)** The preoperative width of palpebral fissure at rest was 9.07 mm in the paralytic side and notably decreased to 8.23 mm after reanimation (*P* < 0.001), closely matched to the healthy width. With forced eye close, the width reduced distinctly from 3.88 mm to 0.14 mm (*P* < 0.001). **(D)** The mean deviation of the philtrum from the hypothetical midline of the face was 3.61 (2.17) mm at rest and 7.74 (2.72) mm when smiling, and distinctly reduced to 1.46 mm and 2.32 mm, respectively, after the transfer and rehabilitation procedure (*P* < 0.001). **(E)** The mean postoperative overall excursion of the oral commissure in the paralytic side was noted to be 5.05 mm, a dramatically great improvement in comparison with preoperative 0.19 mm, slightly less than the excursion of the healthy side. **(F)** At rest, the preoperative oral horizontal excursion was slightly lower in the paralytic side than the healthy side with no statistical significance. Meanwhile, the preoperative mean vertical excursion was 8.30 (3.37) mm in the paralytic side, and markedly elevated to 12.78 (2.91) mm after facial nerve reinnervation, closely matching to the healthy counterpart 12.71 (3.36) mm. Moreover, the overall excursion of oral commissure elevated with limited increment. **(G)** After adjusted by the philtrum deviation, the oral horizontal excursion was greater in the paralytic side than the healthy side with statistical significance, and the difference between the paralytic and healthy side was decreased dramatically from 6.10 (4.27) mm to 2.51 (2.95) mm after surgery. **(H)** A statistically significant augment was found in horizontal (from 21.43 mm to 26.08 mm, *P* < 0.001), vertical (from 10.74 mm to 15.81 mm, *P* < 0.001) and overall excursion (from 24.16 mm to 30.62 mm, *P* < 0.001) when smiling with no significant difference in the excursion of the healthy side. **(I)** After adjusted by the philtrum deviation, the dynamic horizontal excursion of oral commissure was not changed in the paralytic side, while elevated in the healthy side significantly. The difference between the paralytic and healthy side was decreased notably from 9.03 (5.73) mm to 2.59 (5.50) mm after reanimation. **(J)** The angle of the oral commissure was calculated by the arctan of g/h. The mean angle of the oral commissure above the horizontal differed significantly at static and dynamic status between the preoperative and postoperative photos in the paralytic side. **(K)** The angle of slope of bilateral oral commissure was defined by the arctan of (g-g')/(f+f') (g,f: healthy side; g',f': paralytic side). It decreased dramatically from 5.66 (3.90) degrees to 2.49 (2.96) degrees at rest (*P* < 0.001), and from 9.84 (2.86) degrees to 2.24 (3.35) degrees when smiling (*P* < 0.001). **(L)** At rest, the difference of horizontal excursion between the paralytic and healthy side had a discrete distribution around the zero, and tended to concentrate to the zero after facial reanimation. N.S. no statistical difference; *P < 0.05; **P < 0.01; ***P < 0.001.

The Facial Asymmetry Index measured the difference of the paralytic and healthy side in distance between the medial canthus and ipsilateral oral commissure (22) (Figure 3A). Obviously, a lower FAI corresponds to better symmetry of the face, and a bigger reduction of the value indicates better recovery. The oral commissure is not only a landmark of symmetry, but has roles in oral competency, speech, and emotional responses. The FAI and smile excursion were calculated using Photoshop Creative Cloud software (Adobe Systems, USA), and compared for pre- and postoperative photographs at rest (static) and with an attempted smile (dynamic). Briefly, the measurements were calibrated to millimeters using the standardized horizontal white-to-white corneal diameter (11.576 mm) as a reference (31). As illustrated in Figure 3A, the width of palpebral fissure was measured in the line through the center of iris. The perpendicular bisector of the line linking the bilateral central of the iris, which is defined as the central line of the face, intersects with the vermilion margin of the lower lip. The following measurements were carried out referring to the intersection point. The smile excursion was defined as the change in distance from repose to smile of a line linking the reference point to the oral commissure (32). Meanwhile, the angle of the oral commissure relative to the reference point was calculated by the horizontal and vertical distance. The human philtrum is a shallow groove extending from upper lip to nose, and usually seen as the center of the lower face. The width of palpebral fissure, the deviation of the philtrum, and angles or three directional excursions of the oral commissure were calculated and recorded.

### Statistical Analysis

The statistical analysis was performed by GraphPad Prism 7.0 software (GraphPad, USA). Data were presented as the mean ± SD in normally distributed data or as the median (interquartile range, IQR) in ranked data. Two-tailed paired *t* tests were performed to evaluate the significance of preoperative vs. postoperative events or differences between the paralytic and healthy side. Wilcoxon matched-pairs signed rank test was applied to compare the difference of the grade or value acquired by the FNGS2.0 and Sunnybrook FGS. For multivariate analysis, raw data were preprocessed by subtracting the variable mean and dividing by SD, followed by Box-Cox transformation to facilitate modeling. We identified factors for good surgical effectiveness using univariate and multivariate proportional hazard-ratio regression. To investigate the potential predictors for surgical efficacy, we trained a logistic regression model using elastic-net regularization, which helps to improve model robustness. Sociodemographic and clinical variables such as duration, cause, and severity of facial paralysis were included, which could be readily estimated within few minutes. The importance of each predictor to a surgical efficacy metric is calculated by scaling the absolute value of variable coefficients in the logistic model to 0–100. Statistical analysis and visualization were conducted using R-3.6.1 (The R Foundation) with caret, survminer, and ggpubr packages (33, 34). Cox regression was applied to analyze the effect factors of duration of the first facial contraction with biting. Statistically significance: ^*^ *P* < 0.05; ^**^ *P* < 0.01; ^***^ *P* < 0.001.

## Results

The summary of demographics and characteristics of patients is shown in Supplementary Table 1. Twenty patients aged from 18 to 62 years old (median age, 40.5 yr) were included in this study. The median course of facial paralysis was 16.5 months (range, 2–192 month). The most common cause of facial paralysis was tumor-related or surgical complications (10 [50%]), followed by Bell palsy (5 [25%]) and trauma (4 [20%]). The median postoperative follow-up period was 11.8 month (range, 6.6–24.7 month) and the average interval of the first facial contraction while biting was 2.32 month (range, 0.77–5.23 month) after the transfer procedure. Nineteen patients (95%) showed visible activation of the mimetic musculature at the oral commissure postoperatively. One patient caused by purulent otitis media did not show apparent improvement and his smile excursion of the paralytic oral commissure was < 3.0 mm, defining as the minimally smile excursion for good reanimation.

### Facial Nerve Grading Scale 2.0

The patients achieved a preferable symmetry in the lip and nasolabial fold at rest, and they were able to voluntarily elevate the corners of their mouth without severe synkinesis after MFNT. The scores in eye close, nasolabial fold, and oral commissure subdomains decreased significantly after the transfer procedures, while the synkinesis score did not increase distinctly (Figure 2A, Table 1). The grade of facial paralysis decreased except for one patient (Figures 2B,C). In addition, 17 patients were able to close their eyes fully while biting, in comparison with only three patients preoperatively (Supplementary Tables 2, 3).

### Sunnybrook FGS

As illustrated in Figure 2D and Table 1, the patients experienced an improvement in resting and voluntary movement domains (*P* < 0.001), but elevated score in synkinesis domain (*P* < 0.05). Overall, however, only three patients presented moderate synkinesis (obvious but not disfiguring) with an open mouth smile or snarl (Supplementary Table 2). The median total composite score increased from 18 (12.5) to 49 (13.5) after the MFNT, mainly profiting from voluntary movement and resting symmetry (Figure 2D and Table 1), while there was limited score decrement in eye, nasolabial fold, and oral resting symmetry domains. Symmetry in voluntary movement revealed the best functional recovery arose in open mouth smile, snarl, and lip pucker, followed by gentle eye closure, whereas brow lift did not present any improvement (Supplementary Table 2). In fact, no patient had a clinically detectible restoration of brow elevation with biting.

### FaCE Scale

The median preoperative overall score of the FaCE Scale was 24.15 (14.58), and it improved significantly to 59.15 (7.88) after reanimation with elevated scores in all domains (*P* < 0.001, Figure 2E, and Table 1). Even the patient who did not get effective facial movement, from no movement to trace movement, obtained better self-reported score (Supplementary Table 2), indicated that a minimal improvement may be of great importance. Besides, the self-perception of facial disfigurement may change through the reanimation and rehabilitation procedures.

### Restoration of Facial Symmetry

The casual observers are able to detect as little as 3 mm of facial asymmetry of the oral commissure at rest (35). In this study, the preoperative mean FAI was 4.87 ± 2.39 mm at rest, and significantly reduced to 1.46 ± 1.30 mm postoperatively (*P* < 0.001). Furthermore, the FAI with smile decreased from 8.21 ± 2.15 mm to 2.32 ± 2.08 mm after facial reinnervation (*P* < 0.001), below the observer threshold (Figure 3B). The preoperative mean width of palpebral fissure at rest was 9.07 ± 1.58 mm in the affected side, and notably decreased to 8.23 ± 1.23 mm after treatment (*P* < 0.001), closely matched to the nonparalytic width (Figure 3C). With forced eye close, the width decreased distinctly from 3.88 ± 1.62 mm to 0.14 ± 0.41 mm (*P* < 0.001) and 17 patients could close eyes fully with biting (Figure 3C and Supplementary Table 3).

For patients with facial paralysis, it was common that the philtrum and oral commissure deviated to the healthy side. In this series, the mean deviation of the philtrum from the hypothetical midline of the face was 3.61 mm at rest and 7.74 mm when smiling, and reduced to 1.46 mm and 2.32 mm, respectively, after the transfer and rehabilitation procedure (Figure 3D). The mean postoperative overall commissure excursion in the paralytic side was noted to be 5.05 mm, a dramatically great improvement in comparison with preoperative 0.19 mm, but still slightly less than the excursion of the nonparalyzed side (Figure 3E).

The data on oral commissure excursion for all 20 patients are presented in Supplementary Table 3. At rest, the preoperative mean vertical excursion of oral commissure at rest was 8.30 ± 3.37 mm in the affected side, and it was markedly elevated to 12.78 ± 2.91 mm after facial nerve reinnervation, nearly matched to the nonparalytic counterpart (12.71 ± 3.36 mm, Figure 3F). We found that the philtrum deviated to the healthy side in many cases, and the mouth was dragged by the resting tension of the muscle on the healthy side. It attracted our special attention and thoughts that what is the suitable reference when comparing the oral commissure excursion. Therefore, we recalculated the oral horizontal excursion adjusted by the philtrum deviation, and it revealed that the oral horizontal excursion was greater in affected side than the nonaffected side, and the differential value between the two sides was decreased dramatically from 6.10 ± 4.27 mm to 2.51 ± 2.95 mm after surgery (Figure 3G). A significant augment was found in horizontal (from 21.43 to 26.08 mm, *P* < 0.001), vertical (from 10.74 to 15.81 mm, *P* < 0.001), and overall excursion (from 24.16 to 30.62 mm, *P* < 0.01) when smiling (Figure 3H). Obvious oral movement in affected side was observed in 15 patients when smiling after surgery compared with preoperative no or traced movement. Similarly, we recalculated the oral horizontal excursion when smiling adjusted by the philtrum deviation, however, the horizontal excursion did not change in the affected side, but elevated in the nonaffected side significantly (Figure 3I). It was weird but could be explained by the return of the philtrum. Although there was no change in measurement, the affected oral excursion was transformed from passive pulling to active motion. Both oral commissure motion and return of philtrum made a good recovery of the facial movement and aesthetics.

The postoperative angle of the oral commissure above the horizontal differed evidently from the preoperative angle in the paralytic side (Figure 3J). Next, we calculated the angle of slope of bilateral oral commissure, and it decreased dramatically from 5.66 ± 3.90 degrees to 2.49 ± 2.96 degrees at rest (*P* < 0.001), and from 9.84 ± 2.86 degrees to 2.24 ± 3.35 degrees when smiling (*P* < 0.001) (Figure 3K).

### Multivariable Logistic Regression Analysis

**Figure 4:**
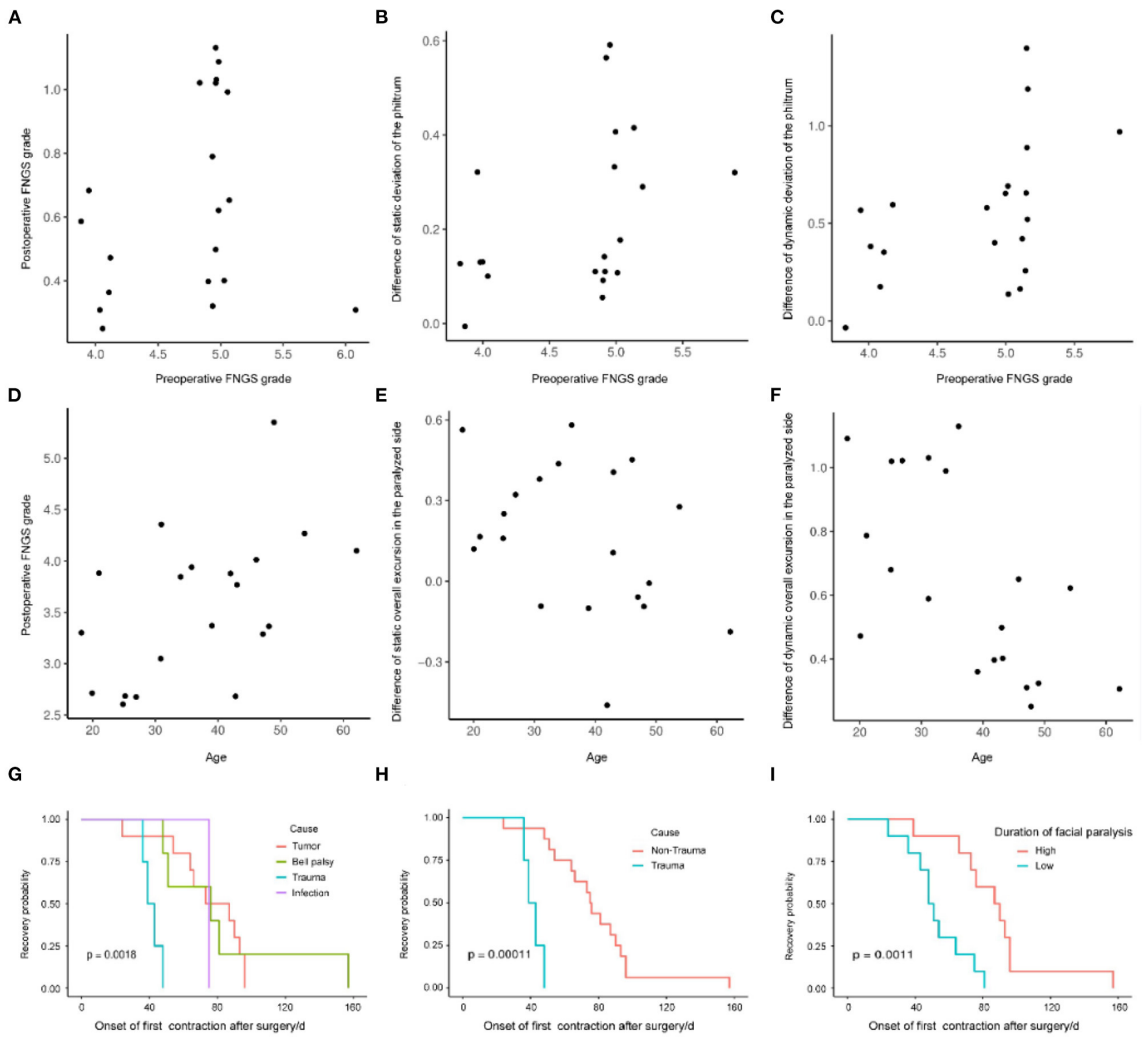
The impact of predictors on the surgical effectiveness by the logistic regression model. **(A–C)** Preoperative FNGS grade was related to merely all scores and metrical data of facial symmetry, such as the postoperative FNGS grade, difference of static and dynamic deviation of the philtrum. **(D–F)** Correlations were observed for the age of the patients with the postoperative FNGS grade, difference of static and dynamic overall excursion in the paralyzed side. **(G–I)** Cox regression revealed that the cause of trauma and shorter duration of facial paralysis were connected with faster recovery.

We evaluated the impact of predictors on surgical effectiveness by the logistic regression model. It showed that age, gender, duration, and cause of facial paralysis, and preoperative FNGS grade were associated with diverse evaluation indices (Table 2). It is worth noticing that preoperative FNGS grade was related to merely all scores and metrical data of facial symmetry, such as the postoperative FNGS grade, difference of static and dynamic deviation of the philtrum (Figures 4A–C), indicating that patients with worse facial paralysis may be more suitable for the MFNT. In addition, correlations were observed for the age of patients with the postoperative FNGS grade, difference of static and dynamic overall excursion in the paralyzed side (Figures 4D–F). Female patients were prone to get higher self-reported FaCE scores. Cox regression found that the cause of trauma and shorter duration of facial paralysis were connected with faster recovery (Figures 4G–I). To sum up, younger patients with severe facial paralysis are preferable to receive the MFNT early for faster and better recovery, especially for traumatic causes.

**Table 2:** Multivariable logistic regression models to predict the effect of nerve transfer by preoperative clinical features.

**Evaluation indices**	**Age**	**Female**	**Duration of facial paralysis**	**Cause trauma**	**Preoperative FNGS grade**
Postoperative FNGS grade	0.20	0	0	−0.17	0.41
Difference of FNGS grade	−0.05	0	−0.03	0	0.12
Difference of composite Sunnybrook score	0	0	0	0.20	0.13
Difference of FaCE score	−0.09	0.49	−0.22	−0.10	0.23
Difference of static FAI	0.23	0	0	0.01	0
Difference of dynamic FAI	−0.32	0.28	−0.21	0.23	0.34
Difference of static overall excursion in the paralyzed side	−0.35	−0.07	−0.08	0	0.19
Difference of dynamic overall excursion in the paralyzed side	−0.73	0	−0.20	−0.13	0.38
Difference of static deviation of the philtrum	0	−0.04	0	0.09	0.21
Difference of dynamic deviation of the philtrum	−0.25	−0.40	0.08	−0.05	0.24
Restoration of dynamic angle of the bilateral oral commissure	−0.11	0	−0.23	0	0.03

### Complications

The surgical complications consisted of minor local infections in one patient. He got good recovery after antibiotic therapy and local rinsing with the saline solution without aggravated scarring. One complained of ocular discomfort with chewing, and no patient had impairment of mastication, temporomandibular joint function, or visible atrophy of masseter.

## Discussions

In this study, the MFNT using the superior trunk of the facial nerve was applied for reanimation in patients with flaccid facial paralysis caused by acoustic neurinoma, dermoid or epidermoid cysts in cerebellopontine angle, Bell palsy, trauma, and bacterial infection. The results demonstrate improvement in static and dynamic symmetry without worsening synkinesis after nerve transfer. In an initial analysis, to avoid bias from the heterogeneity of the scales, we evaluated the severity of facial paralysis by two physician-guided scales and one patient-reported scale. It is recognized that nearly all patients with the worst peripheral facial paralysis could also close the eye partially, which underestimates the severity of paralysis. Otherwise, the elevated score of the FaCE Scale indicated the improvement in facial function, comfort lacrimal control, and social function. In a second analysis, to assess the recovery of symmetry metrically, we compared multiple facial measurement indices before and after facial reanimation. Statistical differences were observed in width of palpebral fissure, deviation of the philtrum, horizontal, vertical, and overall excursion and angles of the oral commissure, demonstrating that patients obtained preferable symmetry after the procedures. The logistic regression revealed that age, gender, duration, and cause of facial paralysis, and preoperative FNGS grade were associated with the effectiveness of the MFNT. In our opinion, younger patients with severe facial paralysis are preferable to receive the MFNT early for faster and better recovery, especially for traumatic causes.

The MFNT has been successfully used to reinnervate paralyzed mimetic muscles in the midface and perioral region. The main reports on the MFNT and related nerve transfer techniques for reanimation of the paralyzed face were summarized in Supplementary Table 4. Hontanilla found that the MFNT resulted in more impressive contraction restoration and shorter recovery time by comparing anastomosis to the hemihypoglossal nerve (14, 18). In this series, the mean duration of first facial contraction onset was 2.32 mo, compatible with the result of Hontanilla (62 d), which is less than the time reported by Albathi (5.6 mo) (14, 22). It can be explained by the high axonal load delivered with the masseteric nerve and proximity to branches of the facial nerve. The histomorphometric analysis demonstrated that the masseteric nerve contained approximately 2,700 myelinated fibers, giving evidence of its usefulness as a source of motor innervation for facial reanimation (27). More importantly, the anatomy of the masseteric nerve is constant and the transfer technique has proved to be less time-consuming requiring no bone dissection. The descending branch of the masseteric nerve can be mobilized easily and sutured directly to selected branches or trunk of the facial nerve. By contrast, the hemihypoglossal-facial neurorrhaphy demands accurate surgical anatomy of the temporal bone and mastoid mobilization to create tension-free sutures avoiding the need of an interposed nerve graft.

The donor-site deficit produced by harvesting the descending branch of the masseteric nerve is minimal. In our cases, no patient had impairment of mastication function or visible atrophy of masseter. Biting abnormalities and temporomandibular joint dysfunction have not been identified at follow-up. Notwithstanding the usefulness of transposition in the reanimation, it is important to emphasize the fact that in this series smile triggering by biting is often necessary to initiate a satisfactory smile, although effortless smile arose in some patients. It is noteworthy that the masseteric nerve demonstrates functional synergy with the facial nerve, as the movement required to activate the nerve by biting goes along much better with contralateral smiling than the movement by pushing the tongue against the teeth, which is necessary to activate the hypoglossal nerve.

The facial asymmetry, particularly of the lower face, triggers significant negative feedback from the observers and adversely affects patients with facial paralysis (4, 5). Restoring facial symmetry both at rest and when smiling is the desired goal in facial reanimation surgery. The reduction in FAI and the difference of adjusted horizontal excursion between the paralytic and healthy side is a reflection of improved tone on the paralyzed side and decreased hypercontraction on the healthy side. At rest on the upper half of the face, around eye, and ectropion are not unusual in the paralyzed side due to the weakened orbicularis oculi muscle and gravity. Restoring blink efficiency is a desirable but challenging goal in facial reanimation with few dynamic solutions. Regenerating axons can reach the orbicularis oculi muscle through the coaptation between the masseteric nerve and the superior trunk of the facial nerve. It was proven by the decreased score in eye domain by FNGS2.0, increased score in symmetry of gentle eye closure in the Sunnybrook FGS, and the elevated eye comfort score in FaCE Scale, which is consistent with reduced width of palpebral fissure at rest and reinforced closing capacity in the paralyzed side. As a result, the periocular synkinesis was presented in some patients, but the benefit overweighed the inherent vice because the synkinesis could be solved by many remedies. Anyway, reinnervation of the orbicularis oculi led to long-lasting improvement in eyelid tone.

The eye-tracking experiment illustrated that attention was preferentially allocated on the mouth region in paralyzed face in repose and it exacerbated with a smile (5, 36). Furthermore, the use of quantitative measurement is beneficial to standardize the severity of facial paralysis, and evaluate the efficacy of the treatment (25, 37, 38). Data showed that improved oral commissure excursion was achieved, and powerful commissure excursion was presented in 19 patients in our series. The deviation of the philtrum and the angle of the oral commissure were restored after the procedures, which revealed the recovery of the lower facial symmetry horizontally and vertically. There was no adequate change in horizontal excursion in resting tone, whereas it became noticeable when the excursion was corrected by the philtrum. Generally, the horizontal excursion of the affected and nonaffected sides was comparative before surgery, while quite differences were presented when smiling. The patients may adjust their oral commissure of the healthy side by dropping or extending it, which contributed to the symmetry at rest. The situation is similar in the postoperative rehabilitation procedures to achieve bilateral coordination (Figures 3G,I,L). These showed that there was some compensation taking place, conscious or unconscious, which restricted or reinforced the movement of the nonparalyzed face to improve the asymmetry.

Synkinesis is a common and disturbing sequela of nerve transfer technique when it involves the lower and upper face (30, 39). In our series, the patients typically reported involuntary eye closure when smiling and clenching. The nerve grafting using the masseteric nerve restores tone to critical zones in the lower facial muscle group, allowing relatively independent movement of the oral commissure with clenching. Similarly, the hemihypoglossal nerve is the most common in anastomoses to the trunk of the facial nerve for facial reanimation, resulting in increased synkinesis and mass facial movements (8, 20). Recent studies revealed that the combined approach decreases synkinesis by providing two separate nerve inputs. Moreover, botulinum toxin therapy and selective neurectomy could be helpful salvage therapy for intolerable or disfiguring synkinesis (40–42).

Given the vital role of facial expression to convey emotional information and the amount of attention focused on the face, it is easy to imagine the negative perception, substantial social and emotional disruption caused by facial paralysis (43). Facial paralysis results in negative self-image, low self-esteem, social distress, depression, and ultimately social isolation (4, 44–46). Although the disfigurement is physical, it is often psychosocial sequelae that are most detrimental. Accordingly, facial reanimation not only restores symmetry and function, but also increases attractiveness and positive facial perception of patients (47). To sum up, the MFNT was proved to be very effective in the reanimation of facial paralysis with advantages of earlier recovery, good facial motor power, the possible spontaneity, and lower complication rate compared to traditional hypoglossal nerve and autogenous nerve transplantation, which was in line with previous findings.

The prediction models help to support tailored clinical decision-making to improve patient outcomes. Sociodemographic and clinical variables were selected because they were thought to be associated with the outcome of the MFNT and could be obtained in a minute. Our results clearly demonstrate that younger patients are preferable to perform the early MFNT for appreciably better and faster recovery. The positive impact of facial nerve reinnervation at the early period on the outcomes is consistent with previous animal or clinical research (10, 14, 16, 22, 48–50). However, some investigators have failed to identify the adverse effect of delaying surgery, even 2 years after facial paralysis (51, 52). Also, it is interesting that the delaying surgical technique using hypoglossal-facial nerve was preferable to immediate repair in preventing synkinesis (53). Based upon our observations, we speculate that the early MFNT using the temporofacial trunk of the facial nerve is an effective technique for facial reanimation, and younger patients with seriously disfiguring facial paralysis caused by traumatic issues benefit more from the procedure. When multiple nerve transfer procedures are feasible, the masseteric nerve should be a preferential choice to optimize outcomes and reduce morbidity.

## Limitations

Several limitations are present in this study. First, a small sample size makes our study less convincible, but the finding of efficacy of the MFNT is clear with clinical importance and highly statistical significance, so further larger confirmatory study is needed. Second, the major disadvantage of reanimation by using motor nerves other than the facial nerve is the potential for dissociation of facial movements and lack of spontaneity. Nevertheless, effortless spontaneous smile has been reported by several authors after the MFNT (13, 15, 54), and supported neuroanatomically with functional MRI and electromyography (55–57), we found that the majority of patients could not produce a smile spontaneously or with no need to clench at all. Finally, the instruments were all used in controlled clinical settings, and the evaluation of spontaneous smile failed. It will be interesting to know how the reanimation technique will fair when tested in real-world settings. Despite these biases, we believe that this study presents powerful evidence for the usage of MFNT with the temporofacial trunk of the facial nerve.

## Conclusions

The MFNT using the temporofacial trunk of the facial nerve is an effective technique for reanimation of the paralyzed midface and perioral region. Satisfactory symmetry and perception can be obtained through the MFNT with limited surgical morbidity. Younger patients with seriously disfiguring facial paralysis are preferable to perform the early MFNT for appreciably better and faster recovery, especially for traumatic causes.

## Data Availability Statement

The raw data supporting the conclusions of this article will be made available by the authors, without undue reservation.

## Ethics Statement

This study was approved by the Institutional Review Board of West China Hospital of Sichuan University. The patients/participants provided their written informed consent to participate in this study. Written informed consent was obtained from the individual(s) for the publication of any potentially identifiable images or data included in this article.

## Author Contributions

TL, YL, and XL: concept and design. TL, YL, SZ, WY, and MZ: acquisition, analysis, or interpretation of the data. TL and XL: drafting of the manuscript. All authors read and approved the manuscript.

## Funding

This work was supported by the grant from Sichuan Province Science and Technology Support Program (2017SZ0006 to YL).

## Conflict of Interest

The authors declare that the research was conducted in the absence of any commercial or financial relationships that could be construed as a potential conflict of interest.

## Publisher's Note

All claims expressed in this article are solely those of the authors and do not necessarily represent those of their affiliated organizations, or those of the publisher, the editors and the reviewers. Any product that may be evaluated in this article, or claim that may be made by its manufacturer, is not guaranteed or endorsed by the publisher.

## Abbreviations

MFNT: masseteric-to-facial nerve transfer
FNGS: Facial Nerve Grading Scale
Sunnybrook FGS: Sunnybrook Facial Grading System
FaCE Scale: Facial Clinimetric Evaluation
FAI: Facial Asymmetry Index.
