# Supplementary material for: Multiple Model Evaluation of the Masseteric-to-Facial Nerve Transfer for Reanimation of the Paralyzed Face and Quick Prognostic Prediction
Source: Front Surg. 2022 Mar 15;9:735231. doi: 10.3389/fsurg.2022.735231 (PMC8964509; doi:10.3389/fsurg.2022.735231)
Supplement: Supplementary file 2 [file Table_4.docx]

**Table S4. Summary of reports on the masseteric-to-facial nerve transfer and related nerve transfer techniques for reanimation of the paralyzed face.**

| First Author, Year | Methods | No. of Patients using only direct MFNT | Total No. of Patients | Main Findings |
| --- | --- | --- | --- | --- |
| Klebuc, 2011[1] | MFNT | 10 | 10 | All patients regained oral competence, good resting tone, and a smile |
| Sforza, 2012[2] | MFNT | 7 | 7 | The significant asymmetry (brow raise, lip purse, smile) reduced after surgery |
| Hontanilla, 2012[3] | MFNT versus hemihypoglossal transposition with a nerve graft | 21 | 46 | Reanimation by both procedures was satisfactory and better symmetry and a faster onset of movement are observed in MFNT with less morbidity |
| Sforza, 2014[4] | MFNT | 14 | 14 | The significant asymmetry of facial movements reduced after surgery |
| Bianchi, 2014[5] | MFNT with and without combined approach | 4 | 60 | All patients are able to smile independent from biting evaluated by the Functional and Aesthetic Grading System. |
| Wang, 2014[6] | MFNT | 16 | 16 | Nine patients had excellent or good function, 5 patients had moderate function, and 2 patients had poor function with only slight synkinetic movements evaluated by Terzis’ Smile Function Evaluation Scale and Mehta’s Synkinesis Evaluation Scale |
| Hontanilla, 2015[7] | MFNT | 6 | 6 | FACIAL CLIMA showed improvement of commissural excursion and velocity. |
| Socolovsky, 2016[8] | MFNT versus hemihypoglossal transposition | 15 | 77 | Hemihypoglossal-to-facial nerve transfers without grafts produce the most satisfactory results, with MFNT providing lesser but still satisfactory outcomes. Hemihypoglossal-to-facial nerve transfers using interposed grafts should likely be avoided whenever possible. |
| Albathi, 2016[9] | MFNT versus hypoglossal transposition | 14 | 19 | Patients can be counseled for facial reanimation surgery as early as 6 months after CPA tumor resection. |
| Hontanilla, 2016[10] | MFNT versus gracilis muscle neurotised to the masseteric nerve | 30 | 66 | For both techniques, women showed significantly earlier and higher rates of spontaneity. |
| Chen, 2017[11] | MFNT | 35 | 35 | The postoperative symmetry scale of the oral commissure at rest improved in 18 of 35 patients. Both oral commissure excursion and position were decreased and dynamic and static symmetry improved after surgery. |
| Ocando, 2019[12] | MFNT or with nerve graft of the great auricular nerve, sural nerve and vestibular nerve. | 3 | 36 | Facial nerve reconstruction offers acceptable functional results in most cases with no significant differences in technical variations. |
| Veen, 2019[13] | MFNT | 71 | 71 | Patients report good voluntary smile with low rates of sequelae. |
| Banks, 2019[14] | MFNT or with deep temporal donor nerve | 39 | 39 | MFNT is a viable option for flaccid facial paralysis patients, and is not reliable in patients with postparalysis facial palsy. |
| Ferraresi, 2021[15] | MFNT with intratemporal translocation of the facial nerve | 11 | 11 | Eight patients had a good to excellent recovery. |
| Leader, 2021[16] | MFNT | 4 | 4 | All patients achieved smile when biting with increased oral commissure excursion. |
| Wang, 2022[17] | MFNT with or without interposition nerve graft | 15 | 32 | MFNT results in strong oral commissure excursion and avoiding obvious synkinesis, while an interposition nerve graft provides better resting symmetry. |
| Roland, 2006[18] | Direct facial-to-hypoglossal neurorrhaphy | 0 | 10 | Nine patients showed strong eyelid and oral sphincter closure and mass motion, facial-hypoglossal neurorrhaphy with parotid release was technically feasible. |
| Manktelow, 2006[19] | Gracilis muscle transfer innervated with the masseter nerve | 0 | 27 | The movement of the upper lip and commissure recovered with satisfying smile and the majority of patients developed the ability to smile spontaneously without jaw movement. |
| Guntinas-Lichius, 2006[20] | Five types of facial nerve reconstructions (direct facial-facial nerve anastomosis, facial nerve interpositional graft, classical hypoglossal-facial nerve anastomosis, hypoglossal-facial nerve interpositional jump graft, and the combined approach) | 0 | 53 | All techniques lead nearly similar and satisfactory results assessed by visual analog scale and May’s facial nerve grading system. Age over 60 years was related to worse outcome. |
| Faria, 2010[21] | MFNT with cross-face sural nerve graft. | 0 | 10 | The procedure improved facial balance at rest, symmetry of the voluntary smile, and lower eyelid function without significantly compromising mastication. |
| Hontanilla, 2013[22] | Free gracilis transplant neurotized to cross-facial nerve graft or ipsilateral masseteric nerve | 0 | 57 | The technique is reliable and provides better symmetry and a higher degree of recovery compared with cross-facial nerve graft neurotization. |
| Rozen, 2016[23] | Nerve grafting with great auricular or sural nerve | 0 | 15 | Grafting provides better resting tone and facial symmetry; however, overall voluntary facial motion is poor. |
| Owusu, 2016[24] | MFNT with cable grafting | 0 | 9 | All patients had return of oral commissure motion with good excursion and minimal synkinesis. |
| Biglioli, 2016[25] | MFNT with nerve graft of the great auricular nerve or sural nerve (with cross-face graft) | 0 | 34 | 31 patients experienced facial nerve function reactivation. The procedure is an efficient surgical technique for early facial reanimation with almost no morbidity. |
| Kochhar, 2016[26] | hypoglossal-to-intratemporal facial nerve transposition | 0 | 17 | Mobilization of the intratemporal facial nerve realizes the direct coaptation to the hypoglossal nerve and is effective in restoring facial tone and symmetry. 16 patients improved in facial symmetry at rest and during animation. |
| Flasar, 2017[27] | Hypoglossal-facial jump nerve suture | 0 | 11 | It had a stronger effect on the face at rest than during movements with improvement of the overall Stennert index and EMG monitoring. |

**References**

1. Klebuc, M.J., *Facial reanimation using the masseter-to-facial nerve transfer.* Plast Reconstr Surg, 2011. **127**(5): p. 1909-15.

2. Chiarella, S., et al., *Facial movement before and after masseteric-facial nerves anastomosis: A three-dimensional optoelectronic pilot study.* Journal of Cranio-Maxillofacial Surgery, 2012. **40**(5): p. 473-479.

3. Hontanilla, B. and D. Marre, *Comparison of hemihypoglossal nerve versus masseteric nerve transpositions in the rehabilitation of short-term facial paralysis using the Facial Clima evaluating system.* Plast Reconstr Surg, 2012. **130**(5): p. 662e-672e.

4. Sforza, C., et al., *Facial reanimation with masseteric to facial nerve transfer: a three-dimensional longitudinal quantitative evaluation.* J Plast Reconstr Aesthet Surg, 2014. **67**(10): p. 1378-86.

5. Bianchi, B., et al., *The masseteric nerve: a versatile power source in facial animation techniques.* Br J Oral Maxillofac Surg, 2014. **52**(3): p. 264-9.

6. Wang, W., et al., *Masseter-to-facial nerve transfer: a highly effective technique for facial reanimation after acoustic neuroma resection.* Ann Plast Surg, 2014. **73 Suppl 1**: p. S63-9.

7. Hontanilla, B. and D. Marre, *Masseteric-facial nerve transposition for reanimation of the smile in incomplete facial paralysis.* Br J Oral Maxillofac Surg, 2015. **53**(10): p. 943-8.

8. Socolovsky, M., et al., *Treatment of complete facial palsy in adults: comparative study between direct hemihypoglossal-facial neurorrhaphy, hemihipoglossal-facial neurorrhaphy with grafts, and masseter to facial nerve transfer.* Acta Neurochir (Wien), 2016. **158**(5): p. 945-57; discussion 957.

9. Albathi, M., et al., *Early Nerve Grafting for Facial Paralysis After Cerebellopontine Angle Tumor Resection With Preserved Facial Nerve Continuity.* JAMA Facial Plast Surg, 2016. **18**(1): p. 54-60.

10. Hontanilla, B. and A. Cabello, *Spontaneity of smile after facial paralysis rehabilitation when using a non-facial donor nerve.* J Craniomaxillofac Surg, 2016. **44**(9): p. 1305-9.

11. Chen, G., et al., *Symmetry Restoration at Rest after Masseter-to-Facial Nerve Transfer: Is It as Efficient as Smile Reanimation?* Plast Reconstr Surg, 2017. **140**(4): p. 793-801.

12. Sanchez-Ocando, M., et al., *Facial nerve repair: the impact of technical variations on the final outcome.* Eur Arch Otorhinolaryngol, 2019. **276**(12): p. 3301-3308.

13. van Veen, M.M., et al., *Patient experience in nerve-to-masseter-driven smile reanimation.* J Plast Reconstr Aesthet Surg, 2019. **72**(8): p. 1265-1271.

14. Banks, C.A., et al., *Five-Year Experience with Fifth-to-Seventh Nerve Transfer for Smile.* Plast Reconstr Surg, 2019. **143**(5): p. 1060e-1071e.

15. Ferraresi, S., et al., *The Masseteric-Facial Anastomosis With Intratemporal Translocation of the Facial Nerve: Step-by-Step Technique and Results.* Oper Neurosurg (Hagerstown), 2021. **21**(5): p. 360-370.

16. Leader, B., et al., *End-to-Trunk Masseteric to Facial Nerve Transfer With Selective Neurectomy for Facial Reanimation.* J Craniofac Surg, 2021. **32**(8): p. 2864-2866.

17. Wang, W.J., et al., *Facial reanimation with interposition nerve graft or masseter nerve transfer: a comparative retrospective study.* Neural Regen Res, 2022. **17**(5): p. 1125-1130.

18. Roland, J., et al., *Direct Facial-to-Hypoglossal Neurorrhaphy with Parotid Release.* Skull Base Surgery, 2006. **16**(2): p. 101-108.

19. Manktelow, R.T., et al., *Smile reconstruction in adults with free muscle transfer innervated by the masseter motor nerve: effectiveness and cerebral adaptation.* Plast Reconstr Surg, 2006. **118**(4): p. 885-99.

20. Guntinas-Lichius, O., M. Streppel, and E. Stennert, *Postoperative functional evaluation of different reanimation techniques for facial nerve repair.* Am J Surg, 2006. **191**(1): p. 61-7.

21. Faria, J.C., G.P. Scopel, and M.C. Ferreira, *Facial reanimation with masseteric nerve: babysitter or permanent procedure? Preliminary results.* Ann Plast Surg, 2010. **64**(1): p. 31-4.

22. Hontanilla, B., D. Marre, and A. Cabello, *Facial reanimation with gracilis muscle transfer neurotized to cross-facial nerve graft versus masseteric nerve: a comparative study using the FACIAL CLIMA evaluating system.* Plast Reconstr Surg, 2013. **131**(6): p. 1241-52.

23. Rozen, S.M., et al., *Intracranial Facial Nerve Grafting in the Setting of Skull Base Tumors: Global and Regional Facial Function Analysis and Possible Implications for Facial Reanimation Surgery.* Plast Reconstr Surg, 2016. **137**(1): p. 267-78.

24. Owusu, J.A., L. Truong, and J.C. Kim, *Facial Nerve Reconstruction With Concurrent Masseteric Nerve Transfer and Cable Grafting.* JAMA Facial Plast Surg, 2016. **18**(5): p. 335-9.

25. Biglioli, F., et al., *Masseteric-facial nerve neurorrhaphy: results of a case series.* J Neurosurg, 2017. **126**(1): p. 312-318.

26. Kochhar, A., et al., *Transposition of the Intratemporal Facial to Hypoglossal Nerve for Reanimation of the Paralyzed Face: The VII to XII TranspositionTechnique.* JAMA Facial Plast Surg, 2016. **18**(5): p. 370-8.

27. Flasar, J., et al., *Quantitative facial electromyography monitoring after hypoglossal-facial jump nerve suture.* Laryngoscope Investig Otolaryngol, 2017. **2**(5): p. 325-330.
